# Quality of Life of Palestinian Patients on Hemodialysis: Cross-Sectional Observational Study

**DOI:** 10.1155/2023/4898202

**Published:** 2023-03-09

**Authors:** Hani H. Naseef, Nadin Haj Ali, Arin Arafat, Sawsan Khraishi, Abdallah Damin AbuKhalil, Ni'meh Al-Shami, Hosniyeh Ladadweh, Mohammad Alsheikh, Abdullah K. Rabba, Imad T. Asmar, Yousef Sahoury

**Affiliations:** ^1^Pharmacy Department, Faculty of Pharmacy, Nursing and Health Professions, Birzeit University, P.O. Box 14, Birzeit, State of Palestine; ^2^Palestine Medical Complex, Ministry of Health, Ramallah, State of Palestine; ^3^Department of Nursing, Faculty of Pharmacy, Nursing and Health Professions, Birzeit University, P.O. Box 14, Birzeit, State of Palestine

## Abstract

**Background:**

Hemodialysis is life-saving and life-altering, affecting patients' quality of life. The management of dialysis patients often focuses on renal replacement therapy to improve clinical outcomes and remove excess fluid; however, the patient's quality of life is often not factored in.

**Objective:**

This study aimed to explore the factors affecting the quality of life of patients on dialysis in Palestine using the Kidney Disease Quality of Life (KDQOL-SFTM) questionnaire.

**Methods:**

A multicenter cross-sectional observational study was conducted at multiple dialysis centers in Palestine, including 271 participants receiving renal replacement therapy. Demographics, socioeconomic, and disease status data were collected. The Arabic version of KDQOL-SFTM was used to assess dialysis patient quality of life. Statistical analysis was performed using SPSS to find correlations among patient factors and the questionnaire's three main domains, the kidney disease component summaries (KDCS), mental component summaries (MCS), and physical component summaries (PCS).

**Results:**

Mean KDCS, MCS, and PCS scores were 59.86, 47.10, and 41.15, respectively. KDC scores were lower among participants aged 40 years or older, with lower incomes, and with diabetes. PCS and MCS scores were lower among patients aged >40, less educated, and lower-income participants. There was a positive correlation between MCS and KDCS (*r* = 0.634, *P*-value <0.001), PCS and KDCS (*r* = 0.569, *P*-value <0.001), as well as MCS and PCS (*r* = 0.680, *P*-value <0.001).

**Conclusion:**

In this study, the KDQOL-SFTM questionnaire revealed lower PCS scores among hemodialysis patients in Palestine. Furthermore, the three domains of the questionnaire were adversely affected by patient income and education status. In addition, physical role, work status, and emotional role showed the lowest scores among the three main domains. Therefore, continuous assessment of patients' quality of life during their journey of hemodialysis using the KDQOL-SFTM along with the clinical assessment will allow the healthcare professionals to provide interventions to optimize their care.

## 1. Introduction

Chronic kidney disease affects more than 10% of the world's population, associated with poor quality of life (QOL) and increased healthcare costs [1]. Kidney failure is a chronic disease in which the kidneys permanently fail to function normally, causing an accumulation of waste products in the blood and tissues, so the patients could be considered potential candidates for life-long renal replacement therapy or a kidney transplant to improve survival [2, 3]. Hemodialysis (HD) a is renal replacement therapy (RRT) procedure that requires hospitalization and is mainly done three times weekly for about 4 hours each session, affecting patients' usual way of living and quality of life [4, 5]. In addition, hemodialysis patients are required to have vascular access through an arteriovenous (AV) fistula, arteriovenous (AV) graft, or a central venous catheter [6]. Furthermore, vascular access is associated with complications such as infections, pain, and hospitalization, significantly impacting patient quality of life [7].

According to the World Health Organization Quality of Life (WHOQOL) assessment, QOL is defined as “individual's perceptions of their position in life in the context of the culture and value systems in which they live and in relation to their goals, expectations, standards, and concerns.” Therefore, the QOL depends on the individual's disease state and physical, mental, and social wellness [8].

HD patients experience poor quality of life (QOL) during different stages of their medical condition [9]. This may be attributed to many factors, including the time spent during the dialysis procedure, access to care, the complications associated with vascular access, and the burden of the disease. Furthermore, overall health, disease status, satisfaction with care, and demographic information affect their quality of life. In addition, the patient's social life, mental health, physical health, and capability to do daily activities can be adversely affected. The quality of life of hemodialysis patients affects patient's survival, hospitalizations, and overall disease outcome [5].

Several studies have been conducted to assess QOL in patients undergoing dialysis. A regional study performed in Tabriz, Iran, showed that parameters such as economic, educational, and occupational status significantly affect the QOL of HD patients. The KDQOL-SF™ was used to assess the QOL in HD patients. Poor QOL was also linked greatly to sleep disturbances. The results showed that poor sleep quality and insomnia are prevalent in HD patients [10]. Another study in Pakistan showed that liquid and food restrictions caused much stress to hemodialysis patients. Many patients were bothered by excessive thirst due to liquid limitations, especially during hot weather [11]. Multiple national studies have been conducted utilizing the Kidney Disease Quality of Life-36 (KDQOL-36) questionnaire in Egypt and Saudi Arabia, and no studies have been conducted in Palestine utilizing this tool [12, 13].

In this study, the KDQOL-SF™ was utilized for the first time in Palestine to assess the QOL of patients with chronic kidney disease on hemodialysis; furthermore, parameters that changed over the years and have not been studied previously in Palestine, such as government support and the availability of dialysis centers, are included in this study.

## 2. Methods

### 2.1. Study Design and Sample

This multicenter cross-sectional observational study was conducted at different HD centers in Palestine. These included the Palestine Medical Complex (Ramallah), Al Hussein Hospital (Beit Jala), Thabit Hospital (Tulkarm), Yasser Arafat Hospital (Salfit), and Alia Hospital (Hebron).

According to the Palestinian Ministry of Health's annual report for 2021, 1570 HD patients are being treated across the West Bank [14]. The Raosoft sample size calculator was used to calculate the sample size for this study with a population size of 1570 patients. The confidence interval was 95%, with a margin of error of 5%. The representative sample size was estimated to be 309 [15].

The study included HM patients who were 18 years and older who receive hemodialysis on regular bases and have received HD for at least one month with a minimum of one session per week. Patients with a physical disability (wheel chaired), patients with an altered level of consciousness, patients on peritoneal dialysis, very sick patients, and those unable to understand either English or Arabic or have incomplete questionnaires were excluded from the study.

### 2.2. Study Tool

The Kidney Disease Quality of Life (KDQOL-SF™) instrument was used in this research [16]. The KDQOL-SF used in this study is a validated tool for patients with kidney disease (KD) and has been translated into different languages [16]. The Arabic version was used with minor modifications. Three physicians and three nurses specializing in managing HD patients reviewed the questionnaire for accuracy, and modifications were made according to the feedback. In addition, a pilot study that included two doctors, three nurses, and ten patients was performed to ensure the usefulness and clarity of the questionnaire before it was administered to the final study population. Based on the pilot study, a final copy of the questionnaire was made with minor modifications.

As described in the supplementary materials, the questionnaire consisted of 44 questions organized into six sections in the following order: patients' demographic information, overall health, information regarding the patient's kidney disease, the effects of kidney disease on the patient's daily life, and the patient's satisfaction with care. (Supplementary materials: Questionnaire Arabic-English combined).

The demographic information section included questions regarding age, sex, marital status, accessibility to a dialysis center, type of insurance, income, employment status, patients' medical history, and other questions regarding HD. Next, the overall health section included the effects of HD on the physical and mental health of the patient. Next, the information regarding the kidney disease section involved questions about the burden of the patient's kidney disease, type of access, cognitive function, and complications associated with HD. The following section included questions about the effects of kidney disease on the patient's daily life, sexual function, sleep, and quality of social interaction. Finally, the patient's satisfaction with care section involved questions regarding the dialysis staff's encouragement and concern about the patient's satisfaction.

The questionnaire covered 19 aspects. These aspects are grouped into three main domains [16]. These domains were the physical health component summary (PCS), mental health component summary (MCS), and kidney disease component summary (KDCS). The PCS scale included the following: physical functioning (10 items), role-physical (4 items), bodily pain (2 items), and general health (5 items); the MCS scale included the following: energy/fatigue (4 items), social functioning (2 items), role emotional (3 items), and emotional well-being (5 items); KDCS scale included the following: symptom/problem list (12 items), effects of kidney disease on daily life (8 items), the burden of kidney disease (4 items), cognitive function (3 items), work status (2 items), sexual function (2 items), quality of social interaction (3 items), sleep (4 items), social support (2 items), dialysis staff encouragement (2 items), and patient satisfaction (1 item).

### 2.3. Statistical Analysis

The data from the different domains were recoded into numeric values of 0 and 100. A value of 0 represents the lowest score which indicates the worst QOL, and a value of 100 represents the highest score which indicates the best QOL. After the scoring process, each scale item was averaged to obtain a scale score. Finally, the mean score for each of the three main domains was calculated [16]. Internal consistency was confirmed for each domain using Cronbach's alpha score. Descriptive statistics were performed, including frequency and percentages for categorical data and means and standard deviations for continuous data. Kolmogorov−Smirnov normality test was conducted to examine the sample distribution. Students' unpaired *t*-tests and one-way ANOVA were conducted to compare the means for normally distributed variables. A *P*-value of 0.05 or less was considered to be statistically significant. In addition, a posthoc test was conducted for the sig. means to determine the association level. Finally, the Pearson Correlation test was performed to assess the association between the three main domains. Analysis was performed using the SPSS software program, version 22.

### 2.4. Ethical Clearance

The Palestinian Ministry of Health provided permission to conduct this study in dialysis units at various centers. The IRB committee of Birzeit University Faculty of Pharmacy, Nursing, and Health Professions granted ethical approval (reference number BZU-PNH-2120). The participants were informed about the study and completed a consent form before participating; consequently, informed consent was obtained from each participant. All methods were performed following the Declaration of Helsinki.

## 3. Results

A total of 348 questionnaires were distributed to HD patients, of which 271 completed the questionnaire (response rate of 78%) since some patients declined to participate due to fatigue and discomfort. Of the total, 184 (67.9%) were aged 40 years or older, and 149 (55%) of the patients were males. Married patients represented 207 (76.4%) of the total. About 122 (45%) of patients had an income of 530–1429 USD, and patients with Bachelor's degrees or higher represented 99 (36.5%) of the total. The main common cause of renal failure was diabetic nephropathy 94 (34.7%) and hypertension 65 (24%). In addition, 106 (39.1%) had been dialyzed for 2-3 years. 180 (66.4%) patients were reported to have dialysis sessions 3 times or more weekly (Table 1).

**Figure 1:**
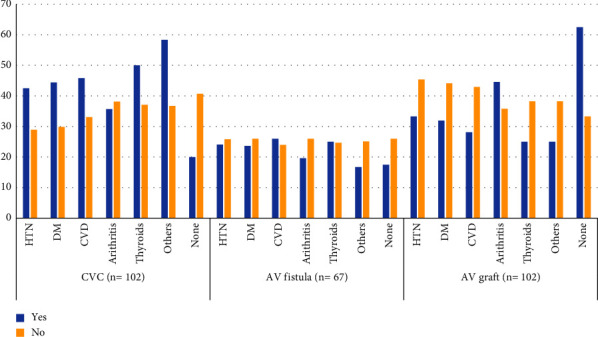
Access port distributions among comorbid patients. (*N* = 271) (CVC: central venous catheter, AV fistula: arteriovenous fistula, AV graft: arteriovenous graft, HTN: hypertension, DM: diabetes mellitus, CVD: cardiovascular diseases).

Figure 1 shows that the CVC access port was more likely to be used among comorbid patients, while the AV graft was the main access port among patients with no comorbidities.

Table 2 shows the mean scores and the Cronbach Alpha values for each level of the three main domains in the instrument. The obtained reliability values had values ranging from 0.554 to 0.943. This indicates that the values ranged from acceptable (general health) to excellent (sexual function). The overall mean scores of the three main components were computed, and the kidney disease component summary (KDCS), mental component summary (MCS), and physical component summary (PCS) were 59.86 (SD 12.62), 47.10 (SD 19.69), and 41.15 (SD 18.78), respectively.

**Figure 2:**
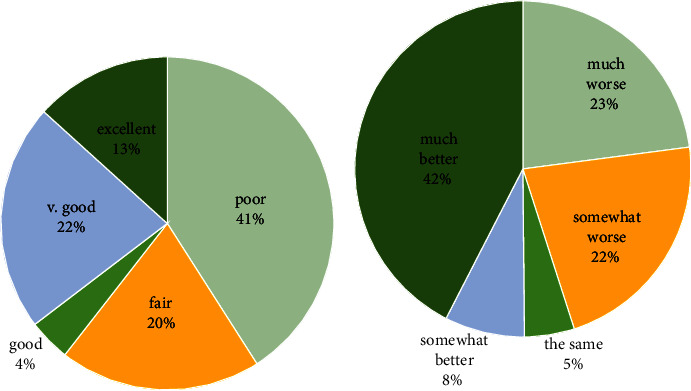
General health evaluation. (a) This year (2022). (b) Compared to the previous year (2021).

In terms of the participant's overall health this year (2022) as compared to the previous year (2021), 41% of patients rated their health to be “poor,” while only 13.3% of them rated their general health to be “excellent” (Figure 2(a)). Referring to Figure 2(b), 42.2% rated their health to be much better than the previous year; meanwhile, about 45% of the patients rated their general health as worse than the previous year.

The impact of age, gender, marital status, educational level, income, cause of kidney disease, duration of dialysis, frequency of dialysis sessions, and site access on the three main domains (KDCS, MCS, PCS) is shown (Table 3). Participants aged 40 years or older had significantly lower mean scores for the KDCS, MCS, and PCS (*P*-value <0.001) than younger participants. The scores were 66.47 ± 11.59, 53.72 ± 19.80, and 48.63 ± 20.38, respectively. Participants with bachelor's degree or higher had significantly higher mean scores regarding MCS (*P*-value 0.003, 53.51 ± 20.63) and PCS (*P*-value <0.001, 47.21 ± 19.15). However, the *P*-value of the KDCS mean score was 0.161, which indicates that educational level had no significant effect on the KDCS score. A total income of 529 USD or lower showed significantly lower mean scores regarding the KDCS (55.60 ± 11.44), MCS (41.33 ± 17.83), and PCS (33.73 ± 18.22) scales upon participants. The *P*-values for the MCS and the PCS mean scores were <0.001, while the KDCS's *P*-values were 0.015. The PCS and KDCS scores were significantly higher for nondiabetic patients. The values were (*P*-value 0.050, 42.78 ± 19.43) and (*P*-value 0.003, 62.13 ± 12.71), respectively. On the other hand, the *P*-value of the MCS score was 0.153, which shows no significant association with the diabetic state among participants. There was no significant effect on scores regarding gender, marital status, duration of dialysis, frequency of dialysis sessions, and access site.

## 4. Discussion

This multicenter observational study assessed the quality of life of hemodialysis patients using the KDQOL-SF questionnaire. The effect of patient demographics, socioeconomic status, finances, education, and comorbidities was evaluated on the questionnaire's three domains. When the scores in the three domains were compared in this study, the lowest score (41.15) was found in the PCS domain, followed by the MCS domain (47.10), while the KDCS domain had the highest score (59.86). Furthermore, the lowest scores were found in physical role (27.58), work status (34.87), and emotional role (37.39). This could be attributed to unemployment, comorbidities, and weekly dialysis sessions. The same results are also seen in a study conducted in Saudi Arabia in 2011 [13]. On the other hand, the highest scores were obtained for social support (81.48) and quality of social interaction (70.60), which may be due to the fact that Palestinians have a very close social life. In a study done in Iran, social support was seen to have the highest score [17].

Participants with a higher level of education showed a significant positive association in the PCS and MCS domains because they have better knowledge of their chronic disease, can cope with it, and thus receive the best possible therapy they can manage. The higher educational level did not significantly affect the KDCS score, which can be attributed to Palestine's lack of jobs and unemployment among all segments of society. The lower income affects the accessibility to the dialysis center and the ability to buy the required medications. Khatib et al. showed in a study conducted in Palestine that a higher educational level is associated with better quality of life. Patients with a higher level of education can better understand their diseases, their complications, and the need to adhere to dialysis sessions [18]. Another similar study in Iran revealed that the level of education positively impacted patients' health and illness due to their accessibility to support and ability to manage the difficulties associated with the disease [17].

It is important to note that a significant proportion of the participants did not have sufficient financial stability and suffered a loss of income during the hemodialysis period. The MCS, PCS, and KDCS scores were significantly lower than those in patients with financial stability, most likely due to life stress associated with decreased financial stability. As a result, patients' quality of life (QOL) deteriorated. Previous studies have also shown that lower-income patients had lower scores for PCS, MCS, and KDCS. The results of this study are consistent with other studies in Romania, which found an association between low economic status and poor QOL [19].

Age significantly affected participants' quality of life. Patients aged 40 years or older had significantly lower PCS, MCS, and KDCS scores because they had more comorbidities and poorer overall health. These findings were also observed in several other studies. For example, a study conducted in Palestine in 2016, which looked at factors affecting patients' quality of life at HD, found that age was one of the most important sociodemographic factors associated with HD-related quality of life [20]. Another study conducted in Palestine in 2015 examining the relationship between treatment satisfaction and health-related quality of life in Palestinian patients with type 2 diabetes mellitus also found that older age was associated with lower health-related quality of life [21].

The PCS and KDCS scores of diabetes mellitus patients were significantly lower than those of nondiabetics, resulting in poorer quality of life in diabetic participants. As mentioned earlier, diabetic nephropathy was the participants' most common cause of renal failure. Therefore, they experience more symptoms of renal disease. In addition, diabetic peripheral neuropathy is a common complication in DM patients. As a result, it affects the physical functioning of these participants by causing significant morbidity (e.g., lower limb amputations) [22]. However, DM did not significantly affect MCS scores. This is probably because some patients are taking antidepressants such as tricyclic antidepressants, gabapentin, and pregabalin to treat neuropathy and increase serotonin levels, which could mask the psychological effects of HD on these patients.

This study showed no statistically significant difference between women and men in the PCS, MCS, and KDCS. Some studies had controversial results. A study published in Saudi Arabia had different results in which male patients had higher scores than females. The author explained that more males than females in their sample were married and earned good income [13]. Another study showed that women had a better QOL score when compared to men due to the difficulty of coping with kidney disease [23]. However, marital status and QOL score in this study were not substantially correlated. This can result from the intimate relationships that Palestinian families maintain. Single patients will not feel alone or helpless as a result.

In this study, no significant statistical difference was observed in access sites with respect to the three domains. A 2018 Pennsylvania article examined the associations between hemodialysis access type and satisfaction with access and health-related quality of life. It suggested that patients with the AV fistula had the greatest satisfaction, leading to a better HRQOL [24].

There was no statistical difference between the duration and frequency of dialysis for the PCS, MCS, and KDCS. Furthermore, a study from Iran published in 2017 showed no significant difference between the duration of dialysis and quality of life in dialysis patients [17]. On the other hand, in other studies, it was hypothesized that the more the time elapsed since the start of dialysis, the better the quality of life and performance. This, in turn, could also impact the clinical parameters of dialysis, such as uremia and anemia symptoms. In addition, patients also become more accustomed to dialysis over time [19, 25] that is hemodialysis.

## 5. Conclusion

In this study, the KDQOL-SF™ questionnaire revealed lower PCS scores among hemodialysis patients in Palestine. Furthermore, the three domains of the questionnaire were adversely affected by patient income and education status. Furthermore, physical role, work status, and emotional role showed the lowest scores among the three main domains. Therefore, continuous assessment of patients' quality of life during their journey of hemodialysis using the KDQOL-SF™ along with the clinical assessment will allow the healthcare professionals to provide interventions to optimize their care.

The strength of this study is the use of a standard instrument (KDQOL-SF™) that allowed the measurement of specific physical and psychological symptoms. A limitation of this study was the face-to-face interview during data collection, which may have influenced patients' responses, as some may not have provided detailed answers for various reasons, and the sample size was less than calculated.

## Figures and Tables

**Table 1:** Baseline characteristics of the study population (*n* = 271).

Variable	Category	Frequency	Percentage
Gender	Female	122	45
	Male	149	55

Age	≥40	184	67.9
	<40	87	32.1

Marital status	Yes	207	76.4
	No	64	23.6

Educational level	Uneducated	21	7.7
	Elementary level	57	21.0
	Secondary level	94	34.7
	Bachelor's degree or high	99	36.5

Income	≤529 USD	100	36.9
	530–1429 USD	122	45.0
	>1429 USD	49	18.1

Comorbidity	None	40	14.8
	HTN	174	64.2
	DM	144	53.1
	CVD	96	35.4
	Arthritis	56	20.7
	Thyroids	12	4.4
	Others	12	4.4

Duration on dialysis	≤1 year	69	25.5
	2-3 years	106	39.1
	≥4 year	96	35.4

HD sessions	≥3 sessions per week	180	66.4
	2 sessions per week	76	28.1
	1 session per week	15	5.5

Access site	Central venous catheter (CVC)	102	37.2
	Arteriovenous fistula (AV fistula)	67	24.7
	Arteriovenous graft (AV graft)	102	37.2

Vaccination	Yes	222	81.9
	No	49	18.1

HTN: hypertension, DM: diabetes mellitus, CVD: cardiovascular diseases, HD: hemodialysis. USD: United States dollar.

**Table 2:** The mean scores for each subject of the 3 mean domains of the KDQOL-SF36 instrument among studied HD (hemodialysis) patients (*N* = 271).

	Number of items	Cronbach *α*	Mean ± SD
*PCS*			**41.15 ± 18.78**
Physical functioning	10	0.925	43.86 ± 29.61
Role-physical	4	0.820	27.58 ± 35.85
Pain	2	0.839	52.51 ± 24.77
General health	5	0.554	40.66 ± 14.94

*MCS*			**47.10 ± 19.69**
Energy/fatigue	4	0.783	42.25 ± 19.57
Social function	2	0.716	53.92 ± 24.49
Role emotional	3	0.773	37.39 ± 39.91
Emotional well being	5	0.788	58.42 ± 20.06

*KDCS*			**59.86 ± 12.62**
Symptoms/problems list	12	0.789	66.03 ± 15.62
Effect of kidney disease on daily life	8	0.608	61.88 ± 17.17
Burden of kidney disease	4	0.707	44.21 ± 20.46
Cognitive function	3	0.722	65.09 ± 20.75
Work status	2	0.636	34.87 ± 40.50
Sexual function	2	0.943	58.89 ± 30.49
Quality of social interaction	3	0.659	70.60 ± 19.40
Sleep	4	0.700	55.57 ± 17.15
Social support	2	0.652	81.48 ± 21.05
Dialysis staff encouragement	2	0.745	63.47 ± 22.48
Patient satisfaction	1	NA	58.73 ± 21.79

PCS: physical health component summary, MCS: mental health component summary, KDCS: kidney disease component summary.

**Table 3:** Quality of life component scores relationship with demographic and clinical variables.

	Physical component score		Mental component score		Kidney disease component score	
	Mean ± SD	*P*-Value	Mean ± SD	*P*-Value	Mean ± SD	*P*-Value
*Age*						
≥40	37.62 ± 16.91	<0.0001	45.29 ± 19.10	0.0001	57.47 ± 12.14	<0.0001
<40	48.63 ± 20.38		53.72 ± 19.80		66.58 ± 11.59	

*Gender*						
Male	42.17 ± 19.42	0.324	48.64 ± 19.14	0.552	60.01 ± 12.86	0.844
Female	39.91 ± 17.96		47.20 ± 20.39		59.60 ± 12.30	

*Marital status*						
Married	39.98 ± 18.14	0.065	47.82 ± 19.45	0.789	59.99 ± 12.41	0.621
Unmarried	44.93 ± 20.40		48.57 ± 20.58		57.83 ± 16.50	

*Educational level*						
Uneducated	39.46 ± 20.55	<0.0001	41.26 ± 20.18	0.003	55.90 ± 11.42	0.161
Elementary level	34.16 ± 14.29		46.74 ± 17.82		59.50 ± 12.01	
Secondary level	39.39 ± 18.72		44.46 ± 18.50		57.78 ± 11.41	
Bachelor's degree or high	47.21 ± 19.15		53.51 ± 20.63		62.36 ± 13.71	

*Income*						
≤529 USD	33.73 ± 18.22	<0.0001	41.33 ± 17.83	<0.001	55.60 ± 11.44	0.015
530–1429 USD	44.28 ± 17.65		51.31 ± 19.87		61.49 ± 12.38	
>1429 USD	48.52 ± 17.84		53.34 ± 19.50		62.45 ± 13.48	

*Cause of KD*						
Diabetic	38.09 ± 17.17	0.050	45.65 ± 18.39	0.153	56.03 ± 11.61	0.003
Non-diabetic	42.78 ± 19.43		49.24 ± 20.29		62.13 ± 12.71	

*Duration of dialysis*						
≤1 year	40.77 ± 18.42	0.757	45.53 ± 20.22	0.207	60.88 ± 13.80	0.798
2-3 years	42.19 ± 18.25		47.12 ± 17.91		59.73 ± 12.92	
≥4 years	40.28 ± 19.73		50.74 ± 21.02		59.08 ± 11.12	

*Frequency of dialysis*						
Once weekly	44.58 ± 20.66	0.485	45.32 ± 19.46	0.480	64.41 ± 8.12	0.326
Twice weekly	42.69 ± 18.20		46.09 ± 19.06		58.71 ± 11.20	
Three times or more per week	40.22 ± 18.89		49.02 ± 19.99		59.96 ± 13.73	

*Access site*						
Central venous catheter	42.60 ± 18.56	0.553	49.96 ± 19.18	0.431	60.63 ± 13.22	0.159
Arteriovenous fistula	39.45 ± 17.95		47.25 ± 20.41		56.48 ± 12.26	
Arteriovenous graft	40.82 ± 19.58		46.52 ± 19.75		61.22 ± 12.14	

KD: kidney disease, USD: United States dollar. positive correlations were found between MCS and KDCS (*r* = 0.634, *P*-value <0.001), PCS and KDCS (*r* = 0.569, *P*-value <0.001), and MCS and PCS (*r* = 0.680, *P*-value <0.001).

## Data Availability

The data used to support the conclusions of this study are provided from the corresponding author upon request.

## Abbreviations

KDQOL-SF: Kidney disease quality of life-short form
KDCS: Kidney disease component summary
MCS: Mental component summary
PCS: Physical component summary
QOL: Quality of life
HD: Hemodialysis
HTN: Hypertension
DM: Diabetes mellitus
CVD: Cardiovascular diseases.

## Additional Points

(i) In addition to assessing therapeutic outcomes, healthcare professionals must pay attention to the quality of life of HD patients. (ii) Patients with kidney disease are affected by several factors, including overall health, disease status, effects on daily activities, care satisfaction, and demographic data. (iii) In order to enhance hemodialysis patients' quality of life (QOL), the factors that impact QOL should be assessed. (iv) This study emphasizes the factors that affect the quality of life of hemodialysis patients across various dialysis clinics in Palestine.

## Supplementary Materials

Supplementary Materials Questionnaire Arabic-English combined consisted of 44 questions organized into six sections in the following order: patients' demographic information, overall health, information regarding the patient's kidney disease, the effects of kidney disease on the patient's daily life, and the patient's satisfaction with care.
