# Supplementary material for: Quality of Life of Palestinian Patients on Hemodialysis: Cross-Sectional Observational Study
Source: ScientificWorldJournal. 2023 Mar 9;2023:4898202. doi: 10.1155/2023/4898202 (PMC10019961; doi:10.1155/2023/4898202)
Supplement: Supplementary Materials — Questionnaire Arabic-English combined consisted of 44 questions organized into six sections in the following order: patients' demographic information, overall health, information regarding the patient's kidney disease, the effects of kidney disease on the patient's daily life, and the patient's satisfaction with care. [file 4898202.f1.pdf]

جامعة بيرزيت
BIRZEIT UNIVERSITY

تقييم جودة الحياة لمرضى غسيل الكلى في فلسطين باستخدام (KDQOL SF^{TM})

- **ما هو الغرض من الدراسة؟**

✔ يتم إجراء هذه الدراسة بالتعاون مع الأطباء ومرضاهم. والغرض من ذلك هو تقييم نوعية حياة مرضى غسيل الكلى.

- **ماذا سيُطلب مني أن أفعل؟**

✔ بالنسبة لهذه الدراسة ، نريدك أن تقوم بإكمال استطلاعًا اليوم حول صحتك ، كيف تشعر وخلفيتك عن الموضوع.

- **سرّية المعلومات؟**

✔ نحن لا نقوم بالسؤال عن اسمك. سيتم دمج إجاباتك مع إجابات المشاركين الآخرين وتسجيلها في نتائج الدراسة. أي معلومات من شأنها أن تسمح بتحديد هويتك ستكون سرية للغاية. بالإضافة إلى ذلك ، سيتم استخدام جميع المعلومات التي تم جمعها فقط لأغراض الدراسة ، ولن يتم الكشف عنها أو استخدامها لأي غرض آخر دون موافقتك المسبقة.

- **كيف سوف استفيد من المشاركة؟**

✔ ستساعدنا المعلومات التي تقدمها على معرفة كيف تشعر حيال رعايتك وتفهمك بشكل أكبر لتأثيرات الرعاية الطبية على صحة المرضى. ستساعد هذه المعلومات أيضاً في تقييم الرعاية المقدمة.

- **هل يجب عليّ المشاركة؟**

✔ لا يتعين عليك ملء الاستبيان ويمكنك رفض الإجابة على أي سؤال. لن يؤثر قرارك بالمشاركة على فرصتك في الحصول على الرعاية.

معلومات ديموغرافية

اسم مركز غسيل الكلى:

1. ما تاريخ اليوم؟

الشهر	اليوم	السنة

2. كم عمرك؟

أقل من 40 سنة	1
40 سنة فما فوق	2

3. ما هو جنسك؟ (ضع/ي دائرة حول إجابة واحدة)

ذكر	1
أنثى	2

4. هل أنت متزوج/ة؟ (ضع/ي دائرة حول إجابة واحدة)

لا	1	الرجاء الانتقال الى السؤال 5
نعم	2	الرجاء الإجابة على السؤال 4(أ)

4.(أ). كم طفلاً لديك؟

عدد الأطفال:	

5. هل يقع مركز غسيل الكلى في مكان سكنك؟ (ضع/ي دائرة حول إجابة واحدة)

يقع في مدينتي/قريتي	1
لا يقع في مدينتي/قريتي	2

6. تَصِل/تَصِلين إلى مركز غسيل الكلى عن طريق: (ضع/ي دائرة حول إجابة واحدة)

المشي	1
سيارة خاصة	2
مواصلات عامة (التكسي مثلاً)	3

7. ما أعلى مستوى تعليمي أكملته؟ (ضع/ي دائرة حول إجابة واحدة)

غير متعلم/ة	1
أساسي	2
ثانوي	3
بكالوريوس فما أعلى	4

8. ما نوع التأمين الصحي لديك؟ (ضع/ي دائرة حول إجابة واحدة)

تأمين حكومي	1
تأمين خاص	2
لا يوجد	3

9. ما دخل الأسرة الكامل؟ (ضع/ي دائرة حول إجابة واحدة)

1850 شيقل أو أقل	1
1851 شيقل -5000 شيقل	2
أكثر من 5000 شيقل	3

10. خلال الأسابيع الأربعة الماضية، هل أنت: (ضع/ي دائرة حول إجابة واحدة)

تعمل/ين بدوام كامل	1
تعمل/ين بدوام جزئي	2
عاطل/ة عن العمل، تبحث/ين عن عمل	3
متقاعد/ة	4
غير قادر/ة على العمل	5
طالب/ة	6
رب/ة منزل	7
لا شيء مما سبق	8

11. هل تعاني/ين من أي أمراض مزمنة؟ (ضع/ي دائرة حول إجابة واحدة)

نعم	1	الرجاء الإجابة على السؤال 11(أ)
لا	2	الرجاء الانتقال إلى السؤال 12

11.(أ). ما هي الأمراض المزمنة التي تعاني/ين منها؟ (ضع/ي دائرة حول إجابة واحدة)

ارتفاع ضغط الدم	1
السكري	2
أمراض القلب والأوعية الدموية	3
التهاب في المفاصل	4
أمراض الغدة الدرقية	5
أخرى (الرجاء التحديد)<br>……………….	6

12. هل تأخذ/ين حالياً أية أدوية موصوفة بوصفة طبية (4 أيام أو أكثر في الأسبوع) بحيث تم وصفها بواسطة طبيب لمعالجة حالة مرضية ما؟ من فضلك لا تقم/تقومي بحساب الأدوية التي لا تحتاج إلى وصفة طبية مثل مضادات الحموضة والأسبيرين. (ضع/ي دائرة على إجابة واحدة)

نعم	1	الرجاء الإجابة على السؤال 12(أ)
لا	2	الرجاء الانتقال إلى السؤال 13

12.(أ). ما هو عدد الأدوية الموصوفة بوصفة طبية التي تتلقاها/تتلقينها حالياً؟

عدد الأدوية	

13. خلال الستة أشهر الماضية، كم يوماً بالمجمل أقمت في المستشفى لمدة ليلة أو أكثر؟ (في حال لم تقضِ أيّ يوم، اكتب/ي 0)

عدد الأيام	

14. خلال الستة أشهر الماضية، كم يوماً بالمجمل تلقيتَ/تِ عناية طبية في المستشفى وعدتَ/تِ إلى المنزل في نفس اليوم؟ (في حال لم تقضِ أيّ يوم، اكتب/ي 0)

عدد الأيام	

15. منذ كم سنة وأنت تقوم/ين بعملية غسيل الكلى؟

عدد السنوات:	

16. كم مرة تقوم/ين بعملية غسيل الكلى خلال الأسبوع الواحد؟ (ضع/ي دائرة حول إجابة واحدة)

مرة	1
مرتين	2
3 مرات أو أكثر	3

17. ما سبب مرض الكلى الذي تعاني منه؟ (ضع/ي دائرة حول جميع الإجابات الممكنة)

لا أعلم	1
ارتفاع ضغط الدم	2
السكري	3
مرض تكيّس الكلى	4
التهاب كبيبات الكلى المزمن	5
التهاب الحويضة والكلية المزمن	6
أخرى (الرجاء التحديد) ………………	7

بالنسبة لصحتك

18. كيف تقيّم/ين صحتك بشكل عام؟ (ضع/ي دائرة حول إجابة واحدة)

ممتازة	1
جيدة جدا	2
جيدة	3
مقبولة	4
ضعيفة	5

19. مقارنة بالسنة الماضية، كيف تُقَدِّر/ين صحتك بشكل عام حالياً؟ (ضع/ي دائرة حول إجابة واحدة)

حالياً أفضل بكثير من السنة الماضية	1
نوعاً ما أفضل من السنة الماضية	2
تقريباً نفس السنة الماضية	3
نوعاً ما أسوأ من السنة الماضية	4
حالياً أسوأ بكثير من السنة الماضية	5

20. النقاط التالية تشمل أنشطة قد تقوم/ين بها في يومك العادي. هل حالتك الصحية حالياً تَحُد من ممارستك لهذه الأنشطة؟ إذا كانت إجابتك نعم، حدد/ي مقدار هذا التأثير؟ (ضع/ي دائرة على خيار واحد من كل سطر)

	نعم، تحدّها كثيراً	نعم، تحدّها قليلاً	لا، لا تحدّها أبداً
أ. أنشطة ذات مجهود عالي، مثل: الركض، وحمل أوزان ثقيلة، والقيام برياضة مجهدة	1	2	3
ب. أنشطة متوسطة، مثل: تحريك طاولة، ودفع مكنسة كهربائية، و لعب البولينغ، و لعب الجولف	1	2	3
ج. حمل المشتريات (البقالة) من السوق	1	2	3
د. صعود أو نزول عدة أدوار(طوابق) باستخدام الدرج	1	2	3
ه. صعود أو نزول دَوْر(طابق) واحد باستخدام الدرج	1	2	3
و. الانحناء، أو الركوع	1	2	3
ز. مشي أكثر من 1 كيلومتر	1	2	3
ح. مشي عدة أحياء	1	2	3
ط. مشي حيّ واحد	1	2	3
ي. الاستحمام أو ارتداء الملابس لوحدك (دون مساعدة أحد)	1	2	3

21. خلال الأسابيع الأربعة الماضية، هل واجهت أي من المشكلات الآتية في عملك أو في أي نشاط يومي آخر بسبب حالتك الصحية؟ (ضع/ي دائرة على خيار واحد من كل سطر)

	نعم	لا
أ. قللتِ الوقت الذي تقضيه/تقضينه في عملك أو في أي نشاط آخر؟	1	2
ب. أنجزت أقل ممّا كنت ترغب/ين به؟	1	2
ج. لم تستطع/تستطيعي إنجاز نفس الأعمال التي كنت تقوم/ين بها سابقاً؟	1	2
ه. شعرت أن الأعمال أو الأنشطة التي تقوم/ين	1	2

بها تحتاج إلى جهدٍ أكبر مقارنة بالسابق؟		

22. خلال الأسابيع الأربعة الماضية، هل واجهت أي من المشكلات الآتية في عملك أو في أي نشاط يومي آخر بسبب حالتك المزاجية (مثل الشعور بالاكتئاب أو القلق)؟ (ضع/ي دائرة على خيار واحد من كل سطر)

	نعم	لا
أ. قللت الوقت الذي تقضيه/تقضينه في عملك أو في أي نشاط آخر؟	1	2
ب. أنجزت أقل ممّا كنت ترغب/ين به؟	1	2
ج. لم تقم/تقومي بالقيام بالأعمال أو الأنشطة بعناية (بتركيز) مقارنةً بالسابق؟	1	2

23. خلال الأسابيع الأربعة الماضية، إلى أي درجة أثرت حالتك الصحية أو المزاجية على أنشطتك الاجتماعية مع عائلتك، أو أصدقائك، أو جيرانك، أو أي مجموعات أخرى؟ (ضع/ي دائرة حول إجابة واحدة)

لم تؤثر أبدا	1
أثرت بشكل قليل	2
أثرت بشكل متوسط	3
أثرت بشكل كبير	4
أثرت بشكل كبير جداً	5

24. في الأسابيع الأربعة الماضية، كم كانت نسبة الألم الجسدي لديك؟ (ضع/ي دائرة حول إجابة واحدة)

لا يوجد ألم أبداً	1
يوجد ألم قليل جداً	2
يوجد ألم بسيط	3
يوجد ألم متوسط	4
يوجد ألم حاد	5
يوجد ألم حاد جداً	6

25. خلال الأسابيع الأربعة الماضية، إلى أي درجة أثّر الألم على عملك المعتاد (داخل وخارج المنزل)؟ (ضع/ي دائرة حول إجابة واحدة)

لم يؤثر أبدا	1
أثر بشكل قليل	2
أثر بشكل متوسط	3
أثر بشكل كبير	4
أثر بشكل كبير جداً	5

26. ستكون الأسئلة القادمة عن كيف كنت تشعر/ين وكيف سارت الأمور معك خلال الأسابيع الأربعة الماضية.
اختر/اختاري الإجابة الأقرب إلى إحساسك. (ضع/ي دائرة على خيار واحد من كل سطر)
كم مرة خلال الأسابيع الأربعة الماضية شعرت بأنك ...

	دائماً	معظم الوقت	تقريباً نصف الوقت	أحياناً	نادرا	أبداً
أ. مفعم/ة بالحيوية والنشاط؟	1	2	3	4	5	6
ب. كنت عصبي/ة ومتوتر/ة جداً؟	1	2	3	4	5	6
ج. محبط/ة لدرجة أن لا شيء يمكنه أن يسعدك؟	1	2	3	4	5	6
د. بحالة من الهدوء والطمأنينة؟	1	2	3	4	5	6
ه. مليء/ة بالطاقة؟	1	2	3	4	5	6
و. كئيب/ة؟	1	2	3	4	5	6
ز. مرهق/ة؟	1	2	3	4	5	6
ح. سعيد/ة؟	1	2	3	4	5	6
ط. متعب/ة؟	1	2	3	4	5	6

27. خلال الأسابيع الأربعة الماضية، كم كانت مدة تأثير حالتك الصحية أو المزاجية على أنشطتك الاجتماعية (مثل: زيارة الأصدقاء والأقارب، الخ...)؟ (ضع/ي دائرة على إجابة واحدة)

طوال الوقت	1
أغلب الوقت	2
أحياناً	3
نادراً	4
لم تؤثر أبداً	5

28. اختر/اختاري الإجابة الأقرب في تحديد مدى صحة كل من الجمل التالية أو خطئها بالنسبة لك؟ (ضع/ي دائرة على خيار واحد من كل سطر)

	أوافق بشدة	أوافق	لا أعلم	لا أوافق	لا أوافق بشدة
أ. يبدو أنني معرض/ة للإصابة بالمرض بسهولة مقارنة بالأشخاص الآخرين	1	2	3	4	5
ب. لا تختلف حالتي الصحية عن أي شخص من حولي	1	2	3	4	5
ج. أتوقع أن تسوء حالتي الصحية	1	2	3	4	5
د. حالتي الصحية ممتازة	1	2	3	4	5

بالنسبة لمرض الكلى الذي تعاني منه

29. ما مدى صحة كل من الجمل التالية أو خطئها بالنسبة لك؟ (ضع/ي دائرة على خيار واحد في كل سطر)

	أوافق بشدة	أوافق	لا أعلم	لا أوافق	لا أوافق بشدة
يؤثر مرض الكلى الذي أعاني منه كثيراً على حياتي	1	2	3	4	5
يمضي الكثير من وقتي في التعامل مع مرض الكلى الذي أعاني منه	1	2	3	4	5
أشعر بالإحباط من التعامل مع مرض الكلى الذي أعاني منه	1	2	3	4	5
أشعر وكأنني عبء على عائلتي	1	2	3	4	5

30. ستكون الأسئلة القادمة عن كيف كنت تشعر/تشعرين وكيف سارت الأمور معك خلال الأسابيع الأربعة الماضية. اختر/اختاري الإجابة الأقرب إلى إحساسك.
كم مرة خلال الأسابيع الأربعة الماضية ...(ضع/ي دائرة على خيار واحد من كل سطر)

	أبداً	نادراً	أحياناً	تقريبا نصف الوقت	معظم الوقت	دائماً
أ. عزلت نفسك عن الأشخاص من حولك؟	1	2	3	4	5	6
ب. كانت ردة فعلك بطيئة تجاه أشياء قيلَتْ أو فُعِلَت؟	1	2	3	4	5	6
ج. تصرفت بتوتر مع من حولك؟	1	2	3	4	5	6
د. شعرت بصعوبة في التفكير أو التركيز؟	1	2	3	4	5	6
ه. شعرت أن علاقتك جيدة مع من حولك؟	1	2	3	4	5	6
و. شعرت أنك مرتبك/ة؟	1	2	3	4	5	6

31. خلال الأسابيع الأربعة الماضية، إلى أي مدى كنت منزعج/ة من كل مما يلي؟ (ضع/ي دائرة على خيار واحد من كل سطر)

	لم أنزعج أبداً	انزعجت إلى حد ما	انزعجت بشكل معتدل	انزعجت كثيرا	انزعجت للغاية
أ. ألم في عضلاتك؟	1	2	3	4	5
ب. ألم في الصدر؟	1	2	3	4	5
ج. تشنجات؟	1	2	3	4	5
د. حكة في الجلد؟	1	2	3	4	5
ه. جفاف في الجلد؟	1	2	3	4	5
و. ضيق في التنفس؟	1	2	3	4	5
ز. ضعف أو دوار(دوخة)؟	1	2	3	4	5
ح. فقدان شهية؟	1	2	3	4	5
ط. إرهاق أو تعب؟	1	2	3	4	5
ي. نمنمة في اليدين أو الأقدام؟	1	2	3	4	5
ك. غثيان أو اضطراب في المعدة؟	1	2	3	4	5

32. ما هو موقع أنبوب سحب الدم المستخدم في حالتك؟ (ضع/ي دائرة حول إجابة واحدة)

الوريد الاصطناعي	1
الناسور الشرياني الوريدي	2
القسطرة الوريدية المركزية	3

33. خلال الأسابيع الأربعة الماضية، إلى أي مدى انزعجت من المشاكل المرتبطة بموقع أنبوب سحب الدم؟ (ضع/ي دائرة حول إجابة واحدة)

لم أنزعج أبداً	1
انزعجت إلى حد ما	2
انزعجت بشكل معتدل	3
انزعجت كثيراً	4
انزعجت للغاية	5

تأثير مرض الكلى على حياتك اليومية

34. ينزعج بعض الأشخاص من تأثير مرض الكلى على حياتهم اليومية، أمّا البعض الآخر لا يتأثر. إلى أي مدى يزعجك مرض الكلى الذي تعاني/ين منه في كل من النقاط التالية؟ (ضع/ي دائرة على خيار واحد من كل سطر)

	لم أنزعج أبداً	انزعجت إلى حد ما	انزعجت بشكل معتدل	انزعجت كثيراً	انزعجت للغاية
أ. تقييد كمية السوائل	1	2	3	4	5
ب. تقييد كمية الطعام	1	2	3	4	5
ج. قدرتك على أداء المهام المنزلية	1	2	3	4	5
د. قدرتك على السفر	1	2	3	4	5
ه. الاعتماد على الطبيب أو أي شخص آخر من الكادر الطبي	1	2	3	4	5
و. القلق أو التعب بسبب مرض الكلى الذي تعاني/ين منه	1	2	3	4	5
ز.الحياة الجنسية	1	2	3	4	5
ح. مظهرك الشخصي	1	2	3	4	5

****الأسئلة الثلاثة القادمة هي أسئلة شخصية حيث تتعلق بنشاطك الجنسي. الإجابات التي سوف تقدمها مهمّة في فهم مدى تأثير مرض الكلى على حياة الشخص.**

35. هل قمت بممارسة أي نشاط جنسي خلال الأسابيع الأربعة الماضية؟ (ضع/ي دائرة على إجابة واحدة)

لا	1	الرجاء الانتقال إلى السؤال 36
نعم	2	الرجاء الإجابة على السؤال 35(أ)

35.(أ). ما مقدار المشكلة التي حدثت في كل ممّا يلي خلال الأسابيع الأربعة الماضية؟ (ضع/ي دائرة على إجابة واحدة من كل سطر)

	لا يوجد مشكلة أبداً	يوجد مشكلة بسيطة	يوجد مشكلة إلى حدٍ ما	يوجد مشكلة كبيرة	يوجد مشكلة شديدة جدا
أ. الاستمتاع بممارسة الجنس	1	2	3	4	5
ب. الوصول للإثارة الجنسية	1	2	3	4	5

****في السؤال القادم، الرجاء تقييم نومك باستخدام مقياس يتراوح بين 0 "سيء جدا" و10 "جيد جداً"**
إذا كنت تعتقد/ين أن وضع نومك يقع في المنتصف بين "سيء جداً" و "جيد جداً" الرجاء وضع دائرة حول الرقم 5. إذا كنت تعتقد/ين أن وضع نومك أفضل بدرجة واحدة من الرقم 5، الرجاء وضع دائرة حول الرقم 6. إذا كنت تعتقد/ين أن وضع نومك أسوء بدرجة واحدة من الرقم 5، الرجاء وضع دائرة حول الرقم 4(وهكذا).

36. على مقياس يتراوح بين 0 و 10، كيف يمكنك تقييم نومك بالمُجمَل؟ (ضع/ي دائرة على رقم واحد فقط)

0	1	2	3	4	5	6	7	8	9	10
سيء جداً										جيد جداً

37. كم مرة خلال الأسابيع الأربعة الماضية ... (ضع/ي دائرة على خيار واحد من كل سطر)

	أبداً	نادراً	أحياناً	تقريباً نصف الوقت	معظم الوقت	دائماً
أ. استيقظت ليلًا ولم تستطع/تستطيعي النوم مجدداً؟	1	2	3	4	5	6
ب. حصلت على قسط كافٍ من النوم؟	1	2	3	4	5	6
ج. واجهت صعوبة بالبقاء مستيقظاً/ةً طوال النهار؟	1	2	3	4	5	6

38. بخصوص عائلتك وأصدقائك، إلى أي مدى أنت راضٍ/راضيةً عن... (ضع/ي دائرة على خيار واحد من كل سطر)

	غير راضٍ أبداً	غير راضٍ	راضٍ قليلاً	راضٍ جداً
أ.كمية الوقت الذي تستطيع/ين قضائه مع عائلتك وأصدقائك ؟	1	2	3	4
ب.الدعم الذي تتلقاه/تتلقينه من قِبَل عائلتك وأصدقائك؟	1	2	3	4

39. خلال الأسابيع الأربعة الماضية، هل عَمِلت في وظيفة مدفوعة الأجر؟ (ضع/ي دائرة حول إجابة واحدة)

نعم	1
لا	2

40. هل وضعك الصحي يمنعك من العمل بوظيفة مدفوعة الأجر؟ (ضع/ي دائرة حول إجابة واحدة)

نعم	1
لا	2

41. كيف تُقَيِّم/تُقَيِّمين صحتك بالمجمل؟ (ضع/ي دائرة حول إجابة واحدة)

0	1	2	3	4	5	6	7	8	9	10
أسوأ ما يُمكِن (سيء لدرجة الموت أو أسوأ)					في المنتصف (بين الأسوأ والأفضل)					أفضل ما يُمكِن

الرضا عن الرعاية

42. عند التفكير في الرعاية الصحية التي تتلقاها/ تتلقينها لمرض الكلى الذي تعاني/ين منه ، إلى أي مدى أنت راضٍ/راضية عن طريقة المعاملة والاهتمام فيك كشخص؟ (ضع/ي دائرة حول إجابة واحدة)

ضعيف جدا	1
ضعيف	2
مقبول	3
جيد	4
جيدجداً	5
ممتاز	6
اكثر من ممتاز	7

43. ما مدى صحة كل من العبارات التالية أو خطئها؟ (ضع/ي دائرة على خيار واحد من كل سطر)

	أوافق بشدة	أوافق	لا أعلم	لا أوافق	لا أوافق
أ. يشجعني طاقم غسيل الكلى على أن أكون مستقلاً قدر الإمكان	1	2	3	4	5
ب. يدعمني طاقم غسيل الكلى في التأقلم مع مرض الكلى الذي أعاني منه	1	2	3	4	5

بالنسبة لڤايروس كورونا (COVID19)

44. هل سبق وأُصِبت بڤايروس كورونا (COVID19)؟

نعم	1
لا	2

45. هل تَلَقَيت تطعيم ڤايروس كورونا (COVID19)؟

نعم	1
لا	2

46. هل قام أحدهم بمساعدتك بتعبئة هذا الاستبيان؟ (ضع/ي دائرة حول إجابة واحدة)

نعم، قام طبيب أو أحد أفراد الطاقم الطبّي بمساعدتي	1
نعم، قام أحد أفراد الأسرة بمساعدتي	2
نعم، قام شخص آخر بمساعدتي	3
نعم، قام الباحثون بمساعدتي	4
لا، لم يساعدني أحد	5

شكرا لك على المشاركة في هذه الدراسة

BIRZEIT UNIVERSITY

STUDY OF QUALITY OF LIFE FOR PATIENTS ON DIALYSIS USING (KDQOL SF™)

- WHAT IS THE PURPOSE OF THE STUDY?

This study is being carried out in cooperation with physicians and their patients. The purpose is to assess the quality of life of patients with kidney disease.

- WHAT WILL I BE ASKED TO DO?

For this study, we want you to complete a survey today about your health, how you feel and your background.

- CONFIDENTIALITY OF INFORMATION?

We do not ask for your name. Your answers will be combined with those of other participants in reporting the findings of the study. Any information that would permit identification of you will be regarded as strictly confidential. In addition, all information collected will be used only for purposes of the study, and will not be disclosed or released for any other purpose without your prior consent.

- HOW WILL PARTICIPATION BENEFIT ME?

The information you provide will tell us how you feel about your care and further understanding about the effects of medical care on the health of patients. This information will help to evaluate the care delivered.

- DO I HAVE TO TAKE PART?

You do not have to fill out the survey and you can refuse to answer any question. Your decision to participate will not affect your opportunity to receive care.

DEMOGRAPHIC INFORMATION

Name of dialysis center:

1. What is today's date?

Month	Day	Year

2. How old are you?

less than 40 years	1
40 years or greater	2

3. What is your gender? (Circle One Number)

Male	1
Female	2

4. Are you currently married? (Circle One Number)

No	1	Please skip to Question 5
Yes	2	Please answer Question 4a

4a. How many children do you have?

Number of Children :	

5. Is the hemodialysis center located in your region? (Circle One Number)

It is located in my country/town	1
It is NOT located in my	2

country/town	

6.You reach the hemodialysis center by… (Circle One Number)

Walking	1
Private Car	2
Public transport (Taxis included)	3

7. What is the highest level of school you have completed? (Circle One Number)

Uneducated	1
elementary level	2
Secondary level	3
Bachelor’s degree or higher	4

8. What kind of health insurance do you have? (Circle One Number)

None	1
Governmental insurance	2
Private insurance	3
Other	4

9. What was your total monthly household income (from all sources) in the LAST CALENDAR YEAR, including yourself, your partner, and others you regard as family who live in your household? (Please remember your answers are confidential.) (Circle One Number)

Less than or equal to 1850 NIS	1
1851 NIS - 5000 NIS	2
More than 5000 NIS	3

10. During the last 4 weeks, were you: (Circle One Number)

Working full-time	1
Working part-time	2
Unemployed, laid off, or looking for work	3
Retired	4
Disabled	5
Student	6
Keeping house	7
None of the above	8

11. Do you suffer from any chronic diseases? (Circle One Number)

Yes	1	Please skip to Question 11a
No	2	Please answer Question 12

11a. What chronic diseases do you suffer from? (Circle All That Apply)

Hypertension (high blood pressure)	1
Diabetes	2
Cardiovascular diseases	3
Arthritis	4
Thyroid gland diseases	5
Other (please specify) ………………….	6

12. Do you currently take prescription medications regularly (4 or more days a week) that are prescribed by your doctor for a medical condition? Please don't count over the counter medications like antacids or aspirin. (Circle One Number)

Yes	1	Please answer Question 12a
No	2	Please skip to Question 13

12a.How many different prescription medications do you currently take?

Number of Medications:	

13. How many days total in the last 6 months did you stay in any hospital overnight or longer? (If none, please write in 0)

Number of Days:	

14. How many days total in the last 6 months did you receive care at a hospital, but came home the same day? (If none, please write in 0)

Number of Days:	

15. For how many years have you been on dialysis?

Number of years:	

16. How many times do you have hemodialysis sessions per 1 week?

Once a week	
Twice a week	
3 times a week or more	

17. What caused your kidney disease? (Circle All That Apply)

Don't know	1
Hypertension (high blood pressure)	2
Diabetes	3
Polycystic Kidney Disease	4
Chronic Glomerulonephritis	5
Chronic Pyelonephritis	6
Other (please specify) ……………….	7

YOUR HEALTH

18. In general, would you say your health is: (Circle One Number)

Excellent	1
Very good	2
Good	3
Fair	4
Poor	5

19. Compared to one year ago, how would you rate your health in general now? (Circle One Number)

Much better now than one year ago	1
Somewhat better now than one year ago	2
About the same as one year ago	3
Somewhat worse now than one year ago	4

Much worse now than one year ago	5

20. The following items are about activities you might do during a typical day. Does your health now limit you in these activities? If so, how much? (Circle One Number on Each Line)

	Yes, limited a lot	Yes, limited a little	No, not limited at all
a. Vigorous activities, such as running, lifting heavy objects, and participating in strenuous sports.	1	2	3
b. Moderate activities, such as moving a table, pushing a vacuum cleaner, bowling, or playing golf.	1	2	3
c. Lifting or carrying groceries.	1	2	3
d. Climbing several flights of stairs.	1	2	3
e. Climbing one flight of stairs.	1	2	3
f. Bending, kneeling, or stooping.	1	2	3
g. Walking more than a kilometer.	1	2	3
h. Walking several blocks.	1	2	3
i. Walking one block.	1	2	3
j. Bathing or dressing yourself.	1	2	3

21. During the past 4 weeks, have you had any of the following problems with your work or other regular activities as a result of your physical health? (Circle One Number on Each Line)

	Yes	No
a. Cut down the amount of time you spent on work or other activities	1	2
b. Accomplished less than you would have liked?	1	2
c. Were you limited in the kind of work or other activities?	1	2

d. Had difficulty performing the work or other activities (for example, it took extra effort)?	1	2

22. During the past 4 weeks, have you had any of the following problems with your work or other regular daily activities as a result of any emotional problems (such as feeling depressed or anxious)? (Circle One Number on Each Line)

	Yes	No
a. Cut down the amount of time you spent on work or other activities?	1	2
b. Accomplished less than you would like?	1	2
c. Didn't do work or other activities as carefully as usual?	1	2

23. During the past 4 weeks, to what extent have your physical health or emotional problems interfered with your normal social activities with family, friends, neighbors, or groups? (Circle One Number)

Not at all	1
Slightly	2
Moderately	3
Quite a bit	4
Extremely	5

24. How much bodily pain have you had during the past 4 weeks? (Circle One Number)

None	1
Very mild	2
Mild	3
Moderate	4
Severe	5

Very severe	6

25. During the past 4 weeks, how much did pain interfere with your normal work (including both work outside the home and housework)? (Circle One Number)

Not at all	1
A little bit	2
Moderately	3
Quite a bit	4
Extremely	5

26. These questions are about how you feel and how things have been with you during the past 4 weeks. For each question, please give the one answer that comes closest to the way you have been feeling. How much of the time during the past 4 weeks . . (Circle One Number on Each Line)

	All of the time	Most of the time	A good bit of the time	Some of the time	A little of the time	None of the time
a. Did you feel full of pep?	1	2	3	4	5	6
b. Have you been a very nervous person?	1	2	3	4	5	6
c. Have you felt so down in the dumps that nothing could cheer you up?	1	2	3	4	5	6
d. Have you felt calm and peaceful?	1	2	3	4	5	6
e. Did you have a lot of energy?	1	2	3	4	5	6

f. Have you felt downhearted and blue?	1	2	3	4	5	6
g. Did you feel worn out?	1	2	3	4	5	6
Have you been a happy person?	1	2	3	4	5	6
Did you feel tired?	1	2	3	4	5	6

27. During the past 4 weeks, how much of the time have your physical health or emotional problems interfered with your social activities (like visiting with friends, relatives, etc.)? (Circle One Number)

All of the time	1
Most of the time	2
Some of the time	3
A little of the time	4
None of the time	5

28. Please choose the answer that best describes how TRUE or FALSE each of the following statements is for you. (Circle One Number on Each Line)

	Definitely True	Mostly True	Don't Know	Mostly False	Definitely False
a. I seem to get sick a little easier than other people.	1	2	3	4	5
b. I am as healthy as anybody I know.	1	2	3	4	5
c. I expect my health to get worse.	1	2	3	4	5
d. My health is excellent.	1	2	3	4	5

YOUR KIDNEY DISEASE

29. How TRUE or FALSE is each of the following statements for you? (Circle One Number on Each Line)

	Definitely True	Mostly True	Don't Know	Mostly False	Definitely False
a. My kidney disease interferes too much with my life.	1	2	3	4	5
b. Too much of my time is spent dealing with my kidney disease.	1	2	3	4	5
c. I feel frustrated dealing with my kidney disease.	1	2	3	4	5
d. I feel like a burden on my family.	1	2	3	4	5

30. These questions are about how you feel and how things have been going during the past 4 weeks. For each question, please give the one answer that comes closest to the way you have been feeling. How much of the time during the past 4 weeks … (Circle One Number on Each Line)

	None of the time	A little of the time	Some of the time	A good bit of time	Most of the time	All of the time
a. Did you isolate yourself from people around you?	1	2	3	4	5	6
b. Did you react slowly to things that were said or done?	1	2	3	4	5	6
c. Did you act irritable toward those around you?	1	2	3	4	5	6

d. Did you have difficulty concentrating or thinking?	1	2	3	4	5	6
e. Did you get along well with other people?	1	2	3	4	5	6
f. Did you become confused?	1	2	3	4	5	6

31. During the past 4 weeks, to what extent were you bothered by each of the following? (Circle One Number on Each Line)

	Not al all bothered	Somewhat bothered	Moderately bothered	Very much bothered	Extremely bothered
a. Soreness in your muscles?	1	2	3	4	5
b. Chest pain?	1	2	3	4	5
c. Cramps?	1	2	3	4	5
d. Itchy skin?	1	2	3	4	5
e. Dry skin?	1	2	3	4	5
f. Shortness of breath?	1	2	3	4	5
g. Faintness or dizziness?	1	2	3	4	5
h. Lack of appetite?	1	2	3	4	5
i. Washed out or drained?	1	2	3	4	5
j. Numbness in hands or feet?	1	2	3	4	5
k. Nausea or upset stomach?	1	2	3	4	5

32. What type of access site is used for your case? (Circle One Number)

Central venous catheter (CVC)	1
Arteriovenous fistula (AV fistula)	2
Arteriovenous graft (AV graft)	3

33. During the past 4 weeks, to what extent were you bothered by problems related to your access site? (Circle One Number)

Not at all bothered	1
Somewhat bothered	2
Moderately bothered	3
Very much bothered	4
Extremely bothered	5

EFFECTS OF KIDNEY DISEASE ON YOUR DAILY LIFE

34. Some people are bothered by the effects of kidney disease on their daily life, while others are not. How much does kidney disease bother you in each of the following areas? (Circle One Number on Each Line)

	Not at all bothered	Somewhat bothered	Moderately bothered	Very much bothered	Extremely bothered
a. Fluid restriction?	1	2	3	4	5
b. Dietary restriction?	1	2	3	4	5
c. Your ability to work around the house?	1	2	3	4	5
d. your ability to	1	2	3	4	5

travel?					
e. Being dependent on doctors and other medical staff?	1	2	3	4	5
f. Stress or worries caused by kidney disease?	1	2	3	4	5
g. Your sex life?	1	2	3	4	5
h. Your personal appearance?	1	2	3	4	5

****The next three questions are personal and relate to your sexual activity, but your answers are important in understanding how kidney disease impacts on people's lives.**

35. Have you had any sexual activity in the past 4 weeks? (Circle One Number)

No	1	Please skip to Question 36
Yes	2	Please answer Question 35a

35a. How much of a problem was each of the following in the past 4 weeks? (Circle One Number on Each Line)

	Not a problem	A little problem	Somewhat of a problem	Very much a problem	Severe problem
a. Enjoying sex?	1	2	3	4	5
b. Becoming sexually aroused?	1	2	3	4	5

**For the following question, please rate your sleep using a scale ranging from 0 representing "very bad" to 10 representing "very good."
If you think your sleep is half-way between "very bad" and "very good," please circle 5. If you think your sleep is one level better than 5, circle 6. If you think your sleep is one level worse than 5, circle 4 (and so on).**

36. On a scale from 0 to 10, how would you rate your sleep overall? (Circle One Number)

0	1	2	3	4	5	6	7	8	9	10
Very Bad										Very Good

37. How often during the past 4 weeks did you… (Circle One Number on Each Line)

	None of the time	A little of the time	Some of the time	A good bit of the time	Most of the time	All of the time
a. Awaken during the night and have trouble falling asleep again?	1	2	3	4	5	6
b. Get the amount of sleep you need?	1	2	3	4	5	6
C. Have trouble staying awake during the day?	1	2	3	4	5	6

38. Concerning your family and friends, how satisfied are you with … (Circle One Number on Each Line)

	Very dissatisfied	Somewhat dissatisfied	Somewhat satisfied	Very satisfied
a. The amount of time you are able to spend with your family and friends?	1	2	3	4
b. The support you receive from your family and friends?	1	2	3	4

39. During the past 4 weeks, did you work at a paying job? (Circle One Number)

Yes	1
No	2

40. Does your health keep you from working at a paying job? (Circle One Number)

Yes	1
No	2

41. Overall, how would you rate your health? (Circle One Number)

0	1	2	3	4	5	6	7	8	9	10
Worst possible (as bad or worse than being dead)					Half-way between worst and best					Best possible health

SATISFACTION WITH CARE

42. Think about the care you receive for kidney dialysis. In terms of your satisfaction, how would you rate the friendliness and interest shown in you as a person? (Circle One Number)

Very poor	1
Poor	2
Fair	3
Good	4
Very good	5
Excellent	6
The best	7

43. How TRUE or FALSE is each of the following statements? (Circle One Number on Each Line)

	Definitely True	Mostly True	Don't Know	Mostly False	Definitely False
a. Dialysis staff encourage me to be as independent as possible.	1	2	3	4	5
b. Dialysis staff support me in coping with my kidney disease.	1	2	3	4	5

REGARDING COVID19

44. Have you been infected with coronavirus (COVID19)? (Circle One Number)

Yes	1
No	2

45. Have you taken the coronavirus vaccine? (Circle One Number)

Yes	1
No	2

46. Did someone help you fill out this survey? (Circle One Number)

Yes, a physician or other health care provider	1
Yes, a family member	2
Yes, someone else	3
Yes, the researchers	4
No	5

THANK YOU FOR TAKING PART IN THIS STUDY
